# Timing of antenatal steroid administration for imminent preterm birth: results of a prospective observational study in Germany

**DOI:** 10.1007/s00404-022-06724-9

**Published:** 2022-08-30

**Authors:** Charlotte Humbeck, Sinje Jonassen, Arne Bringewatt, Mascha Pervan, Achim Rody, Verena Bossung

**Affiliations:** 1 Department of Pediatrics, University Hospital of Würzburg, Würzburg, Germany; 2 Department of Obstetrics and Gynecology, University Hospital of Schleswig-Holstein, Campus Lübeck, Lübeck, Germany; 3 Department of Obstetrics and Gynecology, Westküstenklinikum Heide, Heide, Germany; 4 Department of Obstetrics, University Hospital of Zürich, Zürich, Switzerland

**Keywords:** Antenatal steroids, Corticosteroids, Respiratory distress syndrome, Preterm birth, Prediction

## Abstract

**Purpose:**

To evaluate the timing of antenatal steroid administration and associated medical interventions in women with imminent preterm birth.

**Methods:**

We performed a prospective observational study at a single tertiary center in Germany from September 2018 to August 2019. We included pregnant women who received antenatal steroids for imminent preterm birth and evaluated the interval from administration to birth. 120 women with antenatal steroid application were included into our analysis. Descriptive statistics were performed to analyze factors influencing the timing of antenatal steroids and to evaluate additional medical interventions which women with imminent preterm birth experience.

**Results:**

Of the 120 women included into our study, 35.8% gave birth before 34/0 weeks and 64.2% before 37/0 weeks of gestation. Only 25/120 women (20.8%) delivered within the optimal time window of 1–7 days after antenatal steroid application. 5/120 women (4.2%) only received one dose of antenatal steroids before birth and 3/120 (2.5%) gave birth within 8 to 14 days after antenatal steroids. Most women gave birth more than 14 days after steroid application (72.5%, 87/120). Women with preeclampsia (60%), PPROM (31%), and FGR (30%) had the highest rates of delivery within the optimal time window. Women of all timing groups received additional interventions and medications like antibiotics, tocolytics, or anticoagulation.

**Conclusion:**

Our observational data indicate that most pregnant women do not give birth within 7 days after the administration of antenatal steroids. The timing was best for preterm birth due to preeclampsia, PPROM, and FGR. Especially for women with symptoms of preterm labor and bleeding placenta previa, antenatal steroids should be indicated more restrictively to improve neonatal outcome and reduce untimely and unnecessary interventions.

## What does this study add to the clinical work

**Table:** 

Most pregnant women do not give birth within the optimal time frame of one week after antenatal steroid application. Especially for women with preterm labor and vaginal bleeding due to placenta previa, steroids should be indicated more restrictively to improve neonatal outcome and reduce untimely and unnecessary interventions.	

## Introduction

Preterm birth is affecting 11% of all children born globally [1]. In Germany, the preterm birth rate was 8.36% in 2017 [2]. To improve neonatal morbidity and mortality, international guidelines recommend the application of antenatal steroids (ANS) for pregnant women with imminent preterm delivery before 34 weeks of gestation [2–5]. The improvement of neonatal outcome parameters like respiratory distress syndrome, intraventricular hemorrhage, necrotizing enterocolitis, and death by ANS has already been proven by Liggins and Howie in 1972 [6]. Many other trials have confirmed their findings [7–9]. The glucocorticoids betamethasone and dexamethasone can be used for ANS administration as they cross the placental barrier. The optimal therapeutic effect of ANS has been showed to last for 7 days after a complete course [9, 10] and the therapeutic effect of ANS is significantly reduced after 14 days [11, 12]. This results in a small therapeutic window to achieve the best effect of ANS on neonatal outcome. Thus, a reliable prediction of preterm birth is necessary for the appropriate application of ANS. As preterm birth is the result of multiple pathologies like spontaneous preterm birth due to preterm labor and preterm premature rupture of membranes (PPROM) or medically indicated preterm birth due to maternal preeclampsia or fetal growth restriction (FGR), a precise prediction of preterm birth within the next 7 days is often not possible. Therefore, part of the women who receive ANS for threatened preterm birth never deliver preterm.

Most published trials addressing the timing of steroid application analyze cohorts with preterm delivery after ANS retrospectively or solely evaluate neonatal outcome [11–13]. That approach ignores women who receive ANS but never deliver preterm. Therefore, we chose a prospective design to include all mother–infant pairs who were exposed to ANS. The aim of our study was to prospectively evaluate the timing of ANS with a focus on the group of women who did not deliver within the therapeutic window, nor before 34 weeks but still were exposed to therapies and interventions for suspected imminent preterm birth.

## Materials and methods

### Sample population

We performed a prospective observational study at a tertiary center in Germany from September 2018 to August 2019. We included women who received ANS for suspected imminent preterm birth at our institution. Women under the age of 18 years, women who could not speak German, and women who received ANS outside of our hospital were excluded. After written informed consent was obtained, perinatal data of pregnant women who received antenatal steroids for imminent preterm birth were collected. If patients did not give birth at our hospital, we contacted them by phone to collect perinatal outcome data. We evaluated the ANS–birth interval and classified the patients into four study groups: incomplete ANS (only 1 dose of steroids), optimal timing (2 doses, birth 1 to 7 days after the first dose), suboptimal timing (birth 8 to 14 days after the first dose), and ineffective timing (birth more than 14 days after the first dose). In our hospital, betamethasone (12 mg intramuscular 2 times at an interval of 24 h) was used for ANS as a standard throughout the whole study period. If there was a second cycle of ANS, which was not common (*n* = 1), the time from the last injection of steroids until birth was used. In a second step, we formed two groups for further statistical analysis: the optimal timing group (2 doses, birth 1 to 7 days after first dose) and the non-optimal timing group (only one dose and birth more than 7 days after ANS). The indications for ANS administration were divided into six subgroups: (1) PPROM, (2) preterm labor (including all clinical signs of spontaneous preterm birth except for PPROM), (3) FGR, (4) preeclampsia and HELLP syndrome (including all maternal hypertensive disorders leading to imminent preterm birth), (5) placenta previa/vaginal bleeding, and 6) others (not matching 1–5). The indication for ANS was made by the attending obstetrician. For the preterm labor group, we use a cervical length of 15 mm as a cut-off for indicating ANS based on German guidelines [2] and additionally perform a biomarker test (vaginal placental alpha microglobulin-1, PartoSure®) for cervical lengths between 15 and 25 mm. For FGR, we adhere to national guidelines [14] to indicate delivery as well as antenatal steroid application based on ultrasound, fetal Doppler, and computerized cardiotocography. Descriptive statistics were performed to analyze factors influencing the timing of ANS, the perinatal outcome, and to evaluate the additional medical interventions which women with imminent preterm birth experience. For neonatal birth weight percentiles, we used gender-specific standards for birth weight by gestational age in Germany [15].

### Statistical analyses

Descriptive statistics using percentages for perinatal parameters and corresponding timing of ANS were carried out. For categorical variables Pearson’s Chi-square test or Fisher’s exact test and for continuous variables Mann–Whitney *U* test were used for calculating statistical significance. The type I error level was set to 0.05. A one-way ANOVA was performed to compare the mean ANS–birth intervals for the different indication groups and to compare the mean duration of hospital stay between the four different timing groups. All statistical analyses were performed with SPSS 25.0 software (IBM SPSS Statistics for Windows, Version 25.0. Munich, Germany).

## Results

### Population characteristics

**Fig. 1:**
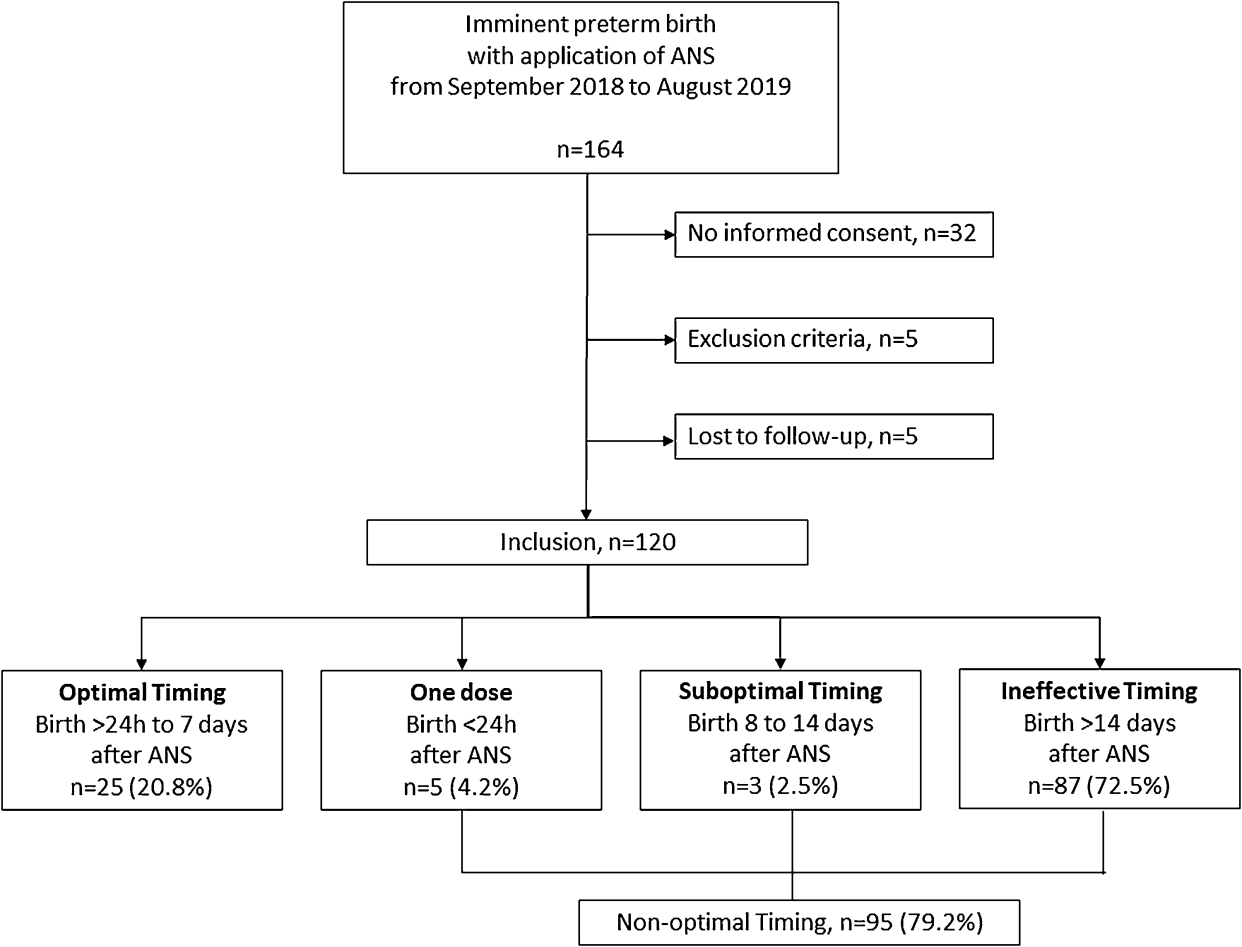
Flowchart of inclusion

Between September 2018 and August 2019, we approached 164 women who received ANS for suspected imminent preterm birth and asked them to participate in the study. 32 women declined consent, 5 women did not meet the inclusion criteria, and 5 women were lost to follow-up as they gave birth in another hospital and could not be contacted. We included 120 mother–infant pairs into our analysis (see Fig. 1). During the same time, 84 women had a live preterm birth between 23/0 and 33/6 weeks of gestation at our hospital. 4.2% of all patients included into our study (*n* = 5/120) received only one dose of betamethasone and gave birth afterward. 20.8% received a complete course of ANS and delivered within the optimal time window of 1–7 days after the first dose and 2.5% gave birth 8–14 days after the first dose of ANS. Most of the patients who received ANS (72.5%) gave birth more than 14 days later.

### Optimal versus non-optimal timing

Based on the described intervals, we formed two groups for further analysis: the optimal timing group with *n* = 25 patients who received a complete course of ANS and delivered within the optimal window of 1–7 days after the first steroid dose and the non-optimal timing group with *n* = 95 patients, which includes the incomplete ANS, the suboptimal ANS, and the ineffective ANS groups (see Fig. 1). 87 of 95 women from the non-optimal group belong to the ineffective group, who gave birth more than 14 days after ANS. The characteristics of the two groups are described in Table 1. There were no statistically significant differences in the baseline characteristics. The gestational age at ANS was comparable in both groups. As expected, the pregnancy outcome differed between the optimal and the non-optimal timing groups (see Table 2). The mean ANS–birth interval was 3.6 days in the optimal timing group versus 47.2 days in the non-optimal timing group (*p* < 0.001). 54.7% of the patients had a preterm birth and 21.1% delivered before 34 weeks of gestation in the non-optimal timing group.

**Table 1:** Cohort characteristics

	Optimal timing *n* = 25	Non-optimal timing *n* = 95	*P* value
Maternal age, mean (SD)	31.4 (3.9)	30.0 (6.1)	0.356^c^
Gravidity, mean (SD)	2.1 (1.9)	2.5 (1.6)	0.084^c^
Parity 1 + , *n* (%)	10 (40.0)	45 (47.4)	0.511^b^
Prior preterm birth, *n* (%)	1 (4.0)	7 (7.4)	1.00^a^
Twin Pregnancy, *n* (%)	6 (24.0)	21 (22.1)	0.840^b^
Smoker, *n* (%)	2 (8.0)	7 (7.4)	1.00^a^
BMI, mean (SD)	26.5 (5.0)	25.0 (6.0)	0.145^c^
Diabetes, *n* (%)	4 (16.0)	23 (19.2)	0.781^a^

Data are given as mean (SD) or *n* (%); *p *values are derived from Fisher’s exact test (^a^) or Pearson’s Chi-square test (^b^) for categorical variables and Mann–Whitney *U *test (^c^) for continuous variables, and the type I error level is set to 0.05. Percentages are given as column percentages

**Table 2:** Indication for antenatal steroid administration and pregnancy outcome

	Optimal timing *n* = 25	Non-optimal timing* n* = 95	*P* value
Indication for ANS			0.003^a^
PPROM, *n* = 16	5 (20.0)	11 (11.6)	
Preterm labor, *n* = 65	7 (28.0)	58 (61.1)	
FGR, *n* = 10	3 (12.0)	7 (7.4)	
Preeclampsia/HELLP, *n* = 10	6 (24.0)	4 (4.2)	
Placenta previa/bleeding, *n* = 7	0	7 (7.4)	
Other indications, *n* = 12	4 (16.0)	8 (8.4)	
Imminent spontaneous preterm birth (PPROM/preterm labor)	12 (48.0)	69 (72.6)	0.019^a^
Pregnancy outcome			
Gestational age at ACS, mean (SD)	29.0 (3.7)	28.7 (3.4)	0.663^b^
Preterm birth < 34/0, *n* (%)	23 (92.0)	21 (22.1)	< 0.001^a^
Preterm birth < 37/0, *n* (%)	25 (100.0)	52 (54.7)	< 0.001^a^
ANS–birth interval, mean days (SD)	3.6 (1.7)	47.2 (29.3)	< 0.001^b^
Gestational age at birth, mean (SD)	29.6 (3.4)	35.5 (3.3)	< 0.001^b^

Data are given as mean (SD) or *n* (%); *p *values are derived from Pearson’s Chi-square test (^a^) for categorical variables and Mann–Whitney *U *test (^b^) for continuous variables, and the type I error level is set to 0.05. Percentages are given as column percentages

The indications for ANS administration were distributed differently between the two groups (see Table 2). In 67.5% of the cases, ANS were given for spontaneous birth aspirations (preterm labor and PPROM), in 32.5% due to threatened medically indicated preterm birth (preeclampsia, FGR, vaginal bleeding, and others). Only 28.0% received ANS because of preterm labor in the optimal timing group versus 61.1% in the non-optimal timing group. Only 7/65 patients who received ANS because of preterm labor gave birth within the optimal window. For placenta previa/bleeding, none of the patients gave birth within the optimal timing. Preeclampsia and HELLP syndrome showed the best timing results.

### ANS–birth intervals by indication groups

**Fig. 2:**
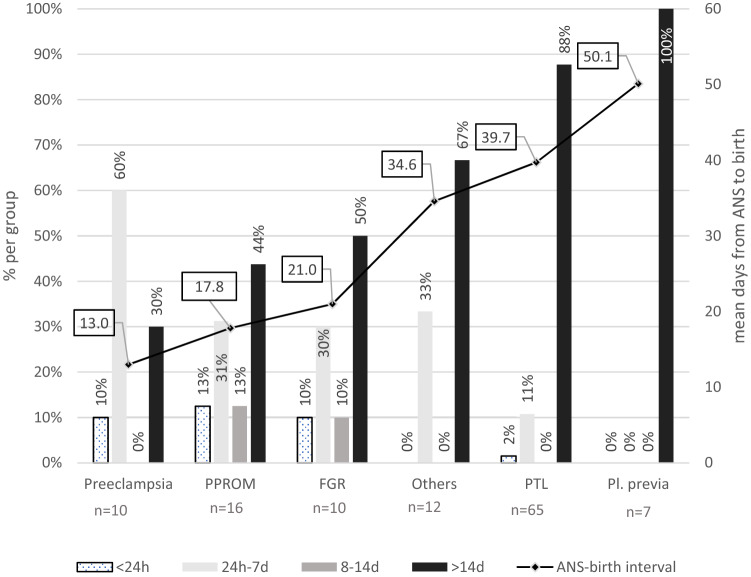
Distribution of ANS–birth interval by indication for ANS. The y-axis gives the indication groups for ANS. The columns give the percentages of the four timing groups for each indication. The line with boxes gives the mean days from ANS to birth for the indication groups

Figure 2 shows the mean days from ANS to birth as well as the timing of ANS according to the six indication groups. Women with preeclampsia had the shortest ANS–birth interval with a mean of 13 days. 60% of them were in the optimal timing group. It was followed by PPROM and FGR with 17.8 and 21 days for the ANS–birth interval. For the largest group of patients with preterm labor (*n* = 65), only 11% gave birth within the optimal time window after ANS and 88% stayed pregnant for more than 14 days after steroids. The mean ANS–birth interval was 39.7 days for this group. The smallest group of placenta previa/bleeding showed the most unfavorable timing. All patients stayed pregnant for more than 14 days after ANS. In a one-way ANOVA, the differences in the mean ANS–birth intervals for the six indication groups reached statistical significance (*p* < 0.001).

### Treatment and outcome characteristics

The overall preterm birth rate of our cohort was 64.2% and 44/120 patients (36.7%) gave birth before 34/0 weeks. 13 patients from the ineffective timing group gave birth before 34 weeks. Their mean GA at ANS was 25.8 weeks and at birth was 30.9 weeks. The mean ANS–birth interval for this group was 37.1 days. Most patients receiving ANS were hospitalized (*n* = 116). The mean total duration of the hospital stay was 11.7 days. 35 patients did not leave the hospital between ANS and birth (29.2%), all others went home after ANS. The mean total duration of the hospital stays for patients who went home between ANS and birth was 9.9 days (SD 11.6). The largest group of pregnant women who delivered more than 14 days after ANS (*n* = 87) stayed in the hospital for a mean of 12.4 days. Most of them went home between ANS and birth (92.0%). Most women who were treated for suspected imminent preterm birth received additional interventions and medications besides ANS: 31.7% were treated with antibiotics, 70.8% with tocolytics, 80.8% with anticoagulation, 16 women were treated with a pessary or cervical stitch cerclage, and 51.7% received vaginal progesterone. The rates of interventions were not statistically different between the study groups except for progesterone, which was most common in the group of patients who gave birth more than 14 days after ANS, as it included a lot of women with preterm labor. Thus, most women from the ineffective group, which did not benefit from ANS, had high rates of interventions.

### Preterm labor group

More than half of the women of our cohort received ANS for suspected preterm labor (*n* = 65, 54.2%). Only 10.7% of them gave birth within the optimal therapeutic window. The mean cervical length at ANS was 13.1 mm. In 38 women, an additional biomarker test was done to evaluate the risk for spontaneous preterm birth (PartoSure®). We found 6 positive and 32 negative test results. None of the patients with a positive biomarker test gave birth within 7 days. Two patients who had a negative result gave birth within 1 week. One was a patient who delivered 7 days after the start of ANS (and the biomarker test) in 25/5 weeks and had a cervical length of 20 mm at admission. The other one gave birth 3 days after the start of ANS (and the biomarker test) in 29/3 weeks with a cervical length of 14 mm at admission. The sensitivity of the biomarker test in our cohort was 0 and the specificity 83.3%. The positive likelihood ratio was 0 and the negative likelihood ratio was 0.01. Concerning the cervicometry, a cut-off value of 15 mm is used in our clinic as a threshold for the indication of ANS. 45 women with preterm labor had a cervical length below 15 mm, and only 5 of them gave birth within 7 days. Of the 20 women with a cervical length of 15 mm or more, 3 gave birth within 1 week. Hence, the 15 mm cut-off value for cervicometry had a sensitivity of 62.5% and a specificity of 29.8% in our cohort. The positive likelihood ratio was 2.17 and the negative likelihood ratio  – 2.06. 52/65 women in the preterm labor group received tocolysis. Only 8 of them gave birth within 14 days (15.4%).

### Neonatal characteristics

The 120 pregnant women of our cohort gave birth to 146 infants as there were 52 twin gestations (see Table 3). 99 infants were born preterm (67.8%) and 56 infants below 34/0 weeks (38.4%). There were 6 perinatal deaths, 3 of them were intrauterine deaths, and 3 children died in the neonatal period. As the infants of the optimal timing group were born at a lower gestational age, their APGAR scores were lower, and they had a higher need for CPAP (continuous positive airway pressure) and intubation.

**Table 3:** Neonatal characteristics

	Total *n* = 146	Optimal Timing *n* = 31	Non-optimal Timing *n* = 115	*P* values
GA at birth, mean (SD)	33.8 (4.26)	29.1 (3.63)	35.1 (3.46)	< 0.001^c^
Preterm birth < 34/0, *n* (%)	56 (38.4)	28 (90.3)	28 (18.0)	< 0.001^b^
Preterm birth < 37/0, *n* (%)	99 (67.8)	31 (100)	68 (59.1)	< 0.001^b^
Male gender, *n* (%), *n* (%)^d^	78 (53.4)	18 (58.0)	60 (52.2)	0.530^b^
Twin gestation, *n* (%)	52 (35.6)	12 (38.7)	40 (34.8)	0.685^b^
Perinatal death (%)	6 (4.1)	4 (12.9)	2 (1.7)	0.019^a^
Birth weight (gram), mean (SD)	2321.2 (918.2)	1414.2 (589.7)	2579.2 (827.8)	< 0.001^c^
Birth weight percentile, mean (SD)	44.0 (26.0)	43.4 (22.3)	44.2 (27.0)	0.971^c^
Length (cm), mean (SD)	45.4 (5.9)	40.4 (5.2)	46.8 (5.4)	< 0.001^c^
Head size (cm), mean (SD)	30.8 (3.6)	27.5 (3.4)	31.8 (3.1)	< 0.001^c^
5-min APGAR, mean (SD)	8.4 (1.4)	7.6 (1.5)	8.6 (1.3)	< 0.001^c^
Umbilical artery pH, mean (SD)	7.26 (0.11)	7.24 (0.16)	7.26 (0.09)	0.826^c^
Umbilical artery BE, mean (SD)	– 4.4 (4.4)	– 4.7 (6.1)	– 4.4 (3.7)	0.380^c^
CPAP, *n* (%)^e^	57 (40.4)	22 (78.6)	35 (31.0)	< 0.001^b^
CPAP (days), mean (SD)^e^	20.4 (22.3)	28.8 (24.8)	15.4 (19.3)	0.020^c^
Intubation, *n* (%)	12 (8.2)	7 (22.6)	5 (4.3)	0.040^a^
Intubation (days), mean (SD)	10.3 (8.3)	15 (8.0)	3.8 (2.1)	0.027^c^

Data are given as mean (SD) or *n* (%); *p* values are derived from Fisher’s exact test (^a^) or Pearson’s chi-square test (^b^) for categorical variables and Mann–Whitney *U *test (^c^) for continuous variables, and the type I error level is set to 0.05. Percentages are given as column percentages

^d^Data available for *n* = 142

^e^Data available for *n* = 141

## Discussion

We performed a prospective observational study including 120 women who received antenatal steroids for imminent preterm birth to evaluate the timing of ANS. Most women (72.5%) gave birth more than 14 days after ANS, resulting in an ineffective timing. Women with preeclampsia, PPROM, and FGR had the highest rates of delivery within the optimal time window. In the largest group of women with signs of preterm labor, only 11% gave birth within the optimal time. Most women treated with ANS were hospitalized and received additional interventions and medications besides ANS like antibiotics, tocolytics, anticoagulation, vaginal progesterone, and pessary or cervical stitch cerclage. Even the mother–infant pairs from the ineffective group, who did not benefit from ANS, had high rates of interventions. Thus, inaccurate prediction of the individual preterm birth risk led to untimely and unnecessary interventions in our cohort.

Antenatal steroids are an important therapeutic tool to reduce neonatal morbidity and mortality in the case of preterm birth [7–9]. Although it is evident that ANS need to be administered within a therapeutic window to achieve a maximum effect for the preterm neonate, our study demonstrates that an optimal timing is challenging for obstetricians. Only 20.8% of the women of our cohort gave birth 1 to 7 days after the first dose of steroids. Five women (4.2%) started with the first dose of ANS but delivered too quickly to finish the treatment cycle. In the clinical setting, a small percentage of women will present with acute labor that cannot be stopped, or with emergency indications for delivery like heavy bleeding or maternal hypertensive disorders. In those cases, it is not possible to finish or even start a cycle of ANS before preterm birth. However, the majority of our cohort delivered more than 14 days after ANS, and 35.8% of them did not even have a preterm birth. This is in line with data from international trials. Most studies had a retrospective design and only included mother–infant pairs who experienced preterm birth below 34 weeks. This study design underestimates the large number of women and infants who stay pregnant beyond 34 weeks and therefore do not benefit from ANS, but are exposed to potential side effects (63.3% of the women in our cohort). Oftentimes, these women receive additional medications like tocolysis, antibiotics, heparin, or progesterone. For example, Levin et al. report an optimal timing of ANS within 24 h to 7 days before preterm birth in 40% of the cases. In their retrospective cohort (*n* = 630), women from the group with hypertensive disorders were most likely to give birth within the optimal time window (62%) [13]. Similarly, in a retrospective single-center study from Sweden (*n* = 431), 41% of the women gave birth within 7 days after ANS administration. Women with preterm labor and PPROM or vaginal bleeding were more likely to give birth within the optimal time window in their cohort, which is contrary to our data. The authors report a higher risk for an adverse neonatal outcome when the ANS–birth interval exceeded 7 days [16]. A retrospective study from the Netherlands (*n* = 1008) included all women, who received ANS during the study period, similar to our design. 45.5% of them gave birth within 7 days, which is higher than in our cohort. The group of women with vaginal bleeding had the lowest rate of deliveries with optimal timing of ANS (13.6%) compared to 61.5% in the group with maternal indications, which is comparable to our data [17]. In their group of women with PPROM, 54.6% of the patients delivered within 1 week after ANS, which is comparable to our PPROM results (44% for incomplete plus optimal timing groups). In their group of women with FGR fetuses, only 37.4% gave birth within 1 week, which also is similar to our cohort (40%). Precise timing of ANS remains a challenge for clinicians leading to a suboptimal effect on neonatal outcome for some mother–infant pairs and overtreatment for others.

To improve the timing of ANS and consecutively improve neonatal outcome, guidelines allow a second cycle of steroids, if the risk for preterm birth continues to be high 7–14 days after the first cycle [2–4]. However, the evidence on repeat ANS cycles is controversial. Therefore, it is not commonly used at our center, only one patient of our cohort received a second cycle of ANS. A 2015 meta-analysis showed that a repeat application of ANS is able to reduce the incidence of respiratory distress syndrome (RR 0.83, 95% CI 0.75–0.91) and a composite serious infant outcome (RR 0.84, 95% CI 0.75–0.95) [18]. Others have showed that the application of a second cycle of ANS does not improve timing. For example, a large retrospective trial by Makhija et al. (*n* = 1356) found no difference in the frequency of delivery within the optimal window after introducing a rescue cycle [19]. Repeat doses can increase known side effects of steroids on the exposed children. These effects include a dose-dependent reduction in birth weight and head circumference [18, 20], and possible associations with neurodevelopmental and behavioral disorders [21, 22]. These side effects are especially critical for the many mother–infant pairs exposed to ANS who do not deliver within the therapeutic window or not preterm at all.

Looking at the neonatal outcome of the two study groups, it might seem surprising at first sight that infants from the optimal timing group had lower APGAR scores as well as a higher need for CPAP and intubation than the non-optimal timing group (see Table 3). This is due to the fact that the largest proportion of the non-optimal timing group gave birth long after their ANS application and the gestational age in this group was significantly higher than in the optimal timing group (35.1 vs. 29.1 weeks, *p* < 0.001). Only 59.1% of the infants were born preterm in the non-optimal timing group versus 100% of the optimal timing group. Hence, the need for respiratory support was a lot lower in the non-optimal timing group.

A reliable prediction of preterm birth is necessary for the appropriate timing of ANS. As preterm birth is a syndrome caused by various conditions, its precise prediction within the next 7 days remains a clinical challenge [23]. For the large group of spontaneous preterm labor, the measurement of the cervical length by vaginal ultrasound and several biomarker tests like fetal fibronectin have been intensively studied to predict preterm delivery [24, 25]. Although a short cervix and biomarkers are able to identify pregnant women at risk for preterm birth, the precise prediction of birth within the next 7 days is not possible [26, 27]. Therefore, many pregnant women receiving ANS for symptoms of preterm labor do not deliver within 1 week. Although we use vaginal ultrasound and biomarker tests for risk assessment at our center, only 10.7% of the women from the preterm labor group of our cohort gave birth within the optimal therapeutic window and 88% delivered more than 14 days after ANS. Clearer criteria for indicating ANS exist for other causes of preterm birth: For example, over 50% of women with PPROM give birth within 1 week [28] increasing the chance for an optimal timing of ANS in this group. For fetuses with FGR, clear criteria from large randomized trials exist to guide appropriate timing of ANS and indicated preterm delivery [29]. In our cohort, the preeclampsia and HELLP syndrome group showed the best results for timing, followed by the FGR group. In the PPROM group, 31% of the patients gave birth within the optimal window and 13% received one dose ANS.

We are aware of strengths and limitations of our study. A major strength is the prospective design, which includes all women receiving ANS even if they did not deliver preterm. Most international trials do not include this group and underestimate the exposed mother–infant pairs who do not benefit from ANS. By choosing the prospective design, we were able to give a complete estimate of the ineffective group. To our knowledge, this is the first analysis of this kind for a German population. We are aware of some limitations. One limitation is the relatively small cohort size, and a second one is the single-center design of our study. As standards of care and patient characteristics differ between regions and hospitals, our results may not be fully transferable to other populations, although they are in line with international data.

## Conclusions

Our observational data indicate that most pregnant women do not give birth within 7 days after the administration of antenatal steroids. The timing was best for preterm birth due to preeclampsia, PPROM, and FGR. Especially, for the large group of women with symptoms of preterm labor and women with bleeding due to placenta previa, antenatal steroids should be indicated more restrictively to improve neonatal outcome and reduce untimely and unnecessary interventions.

## Abbreviations

ANOVA: Analysis of variance
ANS: Antenatal steroids
BMI: Body mass index
CPAP: Continuous positive airway pressure
FGR: Fetal growth restriction
HELLP: Hemolysis, elevated liver enzymes, low platelets
PPROM: Preterm premature rupture of membranes
PTL: Preterm labor
SD: Standard deviation
